# Exploration of factors associated with turnaround time when evaluating non-tuberculous mycobacterium cultures

**DOI:** 10.3389/fmed.2026.1766817

**Published:** 2026-02-26

**Authors:** Xingxing Lou, Sheng Zhao, Sipei Wang, Shanshan Jin, Tinghua Ye, Xinling Pan

**Affiliations:** 1 Department of Clinical Laboratory, Wenzhou Medical University Affiliated Dongyang Hospital, Dongyang, Zhejiang, China; 2 Department of Biomedical Sciences Laboratory, Wenzhou Medical University Affiliated Dongyang Hospital, Dongyang, Zhejiang, China

**Keywords:** acid-fast bacilli, clinical laboratory, culture, non-tuberculous mycobacteria, rapidly growing mycobacteria, slow-growing mycobacteria, turnaround time

## Abstract

**Background:**

A positive culture of non-tuberculous mycobacteria (NTM) is a key diagnostic criterion for NTM disease. Due to the slow growth rate of NTM, the turnaround time (TAT) for culture specimens is often lengthy, posing significant challenges for the diagnosis and treatment of related diseases. This study aimed to explore factors influencing TAT in NTM culture testing, assess its potential clinical value, and identify ways to expedite clinical decision-making.

**Methods:**

NTM identified by *HSP65* sequencing in a tertiary hospital from June 2022 to May 2024 were retrospectively included, and patients’ data were collected. TAT was defined as the time between specimen receipt and report issuance. Differences in TAT between groups were analyzed using the rank-sum test. Correlations were evaluated using Spearman’s correlation analysis, and a generalized linear model was applied to identify independent factors influencing TAT.

**Results:**

A total of 289 NTM strains were finally enrolled in this study, including rapidly growing mycobacteria (RGM, *n* = 22) and slow-growing mycobacteria (SGM, *n* = 267). The median TAT for RGM was 7 days, while the median TAT for SGM was 11 days, indicating a statistically significant difference (*p* < 0.001). Representative species within RGM and SGM also showed significant TAT discrepancies. Patients with NTM pulmonary disease, bronchiectasis, or cavitation had shorter median TATs (8–9 days) than those without such findings (*p* < 0.05). TAT showed a negative correlation with acid-fast bacilli smear grades (correlation coefficient: −0.490, *p* < 0.001), and the acid-fast bacilli smear result was confirmed as the only determinant of TAT in the final regression model (Wald *χ*^2^ = 39.71, *p* < 0.001).

**Conclusion:**

TAT for NTM culture was significantly influenced by species category, with RGM showing shorter TATs than SGM. Variations in TAT were observed among different species within the same complex. Additionally, TAT was associated with clinical diagnoses and imaging findings. The acid-fast bacilli smear result was the only independent factor affecting TAT, which could help to guide clinical workflow optimization.

## 1 Introduction

Non-tuberculous mycobacteria (NTM) refer to mycobacteria other than members of the *Mycobacterium tuberculosis* complex and *Mycobacterium leprae* (1). NTM are classified into rapidly growing mycobacteria (RGM) and slow-growing mycobacteria (SGM) (2). Further categorization includes specific complexes or groups, such as the *Mycobacterium avium* complex (MAC), *Mycobacterium abscessus* complex (MABC), and *Mycobacterium simiae* complex (MSC) (3).

NTM are opportunistic pathogens that can cause lesions in various tissues and organs, collectively referred to as NTM disease. Among these, NTM pulmonary disease (NTM-PD) is the most common and is increasing in incidence worldwide (4). In some countries, NTM infection rates currently exceed those of the *M. tuberculosis* complex (5). Therefore, efficient isolation and detection of NTM are essential. In the majority of hospitals, particularly primary care facilities, NTM detection relies on conventional mycobacterial culture. Due to the slow growth of NTM, it often takes several days or weeks for laboratories to issue reports, resulting in prolonged turnaround time (TAT). This delay poses significant challenges for the timely diagnosis and treatment of NTM-related diseases. TAT, defined as the interval from specimen receipt to report delivery (6), is a widely used metric for assessing laboratory efficiency (7, 8). However, few studies have explored TAT specifically for NTM.

Previous studies have shown that the time to positive (TTP) in mycobacterial culture is negatively correlated with acid-fast bacilli (AFB) smear grades and colony numbers (9). In addition, TTP has the potential to be a predictive biomarker for *M. avium* pulmonary disease (10). Unlike TTP, TAT encompasses the entire process from specimen receipt to report issuance, providing a more comprehensive metric. Despite its significance, the impact of various NTM species and host factors on TAT remains poorly understood, prompting this study to investigate these relationships.

## 2 Materials and methods

### 2.1 Mycobacterial culture and reporting process

Upon receipt, mycobacterial culture specimens were stored at 4 °C and processed the following morning. Isolation and culture were performed according to standard operating procedures, and the BACTEC MGIT 960 system (BD, USA) was used to detect mycobacteria. Once the instrument reported a positive result, Ziehl–Neelsen staining was performed. For specimens with positive AFB results, MPB64 antigen detection (Chuangxinshengwu, Hangzhou, China) was used for mycobacterial identification. If the MPB64 antigen test was negative, the obtained strains were reported as NTM.

### 2.2 NTM species identification

Specimens with AFB-positive microscopy but negative MPB64 antigen tests were retained for further NTM species identification. After centrifugation at 10,000 *g* for 5 min and washing with 500 μL of phosphate-buffered solution (PBS), 200 μL of Tris-EDTA buffer was added, followed by heating at 100 °C for 10 min to prepare the template for polymerase chain reaction (PCR) amplification. The *HSP65* gene was amplified, after which the amplification products were verified by gel electrophoresis and processed for Sanger sequencing. Sequences were analyzed using NCBI BLAST, with the highest scoring match being identified as the strain’s species, as described in our previous study (11).

### 2.3 Definition of TAT

TAT was defined as the time from specimen receipt to NTM report issuance. It included pre-processing, mycobacteria culture, AFB smear, MPB64 antigen testing, and final result delivery (12).

### 2.4 Data collection

NTM strains were collected between June 2022 and May 2024. For serial strains of the same species from the same patient during a single hospitalization, the mean TAT was used. Collected data included patient demographics, comorbidities, medical history, imaging findings, and laboratory results.

### 2.5 Statistical analyses

Statistical analyses were performed using SPSS version 26.0. As TAT was non-normally distributed, results were expressed as medians and quartiles. The rank-sum test was used to assess group differences, and Spearman’s correlation analysis was used to assess correlations. The threshold for statistical significance was set at a *p*-value of < 0.05. Based on the univariate analysis, variables with statistical significance (*p* < 0.05) or clinical relevance were selected for inclusion in the generalized linear model with a gamma distribution and log link function, to identify independent factors associated with TAT.

## 3 Results

### 3.1 NTM species identification

A total of 289 NTM strains were included in the study, comprising RGM (*n* = 22) and SGM (*n* = 267). The majority were MAC (*n* = 244, 84.4%), followed by MABC (*n* = 21, 7.3%) and MSC (*n* = 6, 2.1%) (Table 1). Within MAC, the most prevalent species were *M. chimaera* (*n* = 124), *M. intracellulare* (*n* = 65), and *M. colombiense* (*n* = 33).

**Table 1:** Distribution and number of strains.

Mycobacterial complexes	Species	Count
MAC (*n* = 244)	*Mycobacterium chimaera*	124
	*Mycobacterium colombiense*	33
	*Mycobacterium intracellulare*	65
	*Mycobacterium avium subsp. hominissuis*	3
	*Mycobacterium marseillense*	6
	*Mycobacterium yongonense*	5
	*Mycobacterium avium*	8
MABC (*n* = 21)	*Mycobacterium abscessus*	14
	*Mycobacterium massiliense*	7
MSC (*n* = 6)	*Mycobacterium lentiflavum*	4
	*Mycobacterium paraense*	2
Others (*n* = 18)	*Mycobacterium gordonae*	3
	*Mycobacterium seoulense*	3
	*Mycobacterium paragordonae*	2
	*Mycobacterium kansasii*	2
	*Mycobacteria fortuitum*, *Mycobacteria farcinogenes*, etc.	8

MAC, *Mycobacterium avium* complex; MABC, *Mycobacterium abscessus* complex; MSC, *Mycobacterium simiae* complex.

### 3.2 TAT distributions for different NTM species

**Figure 1:**
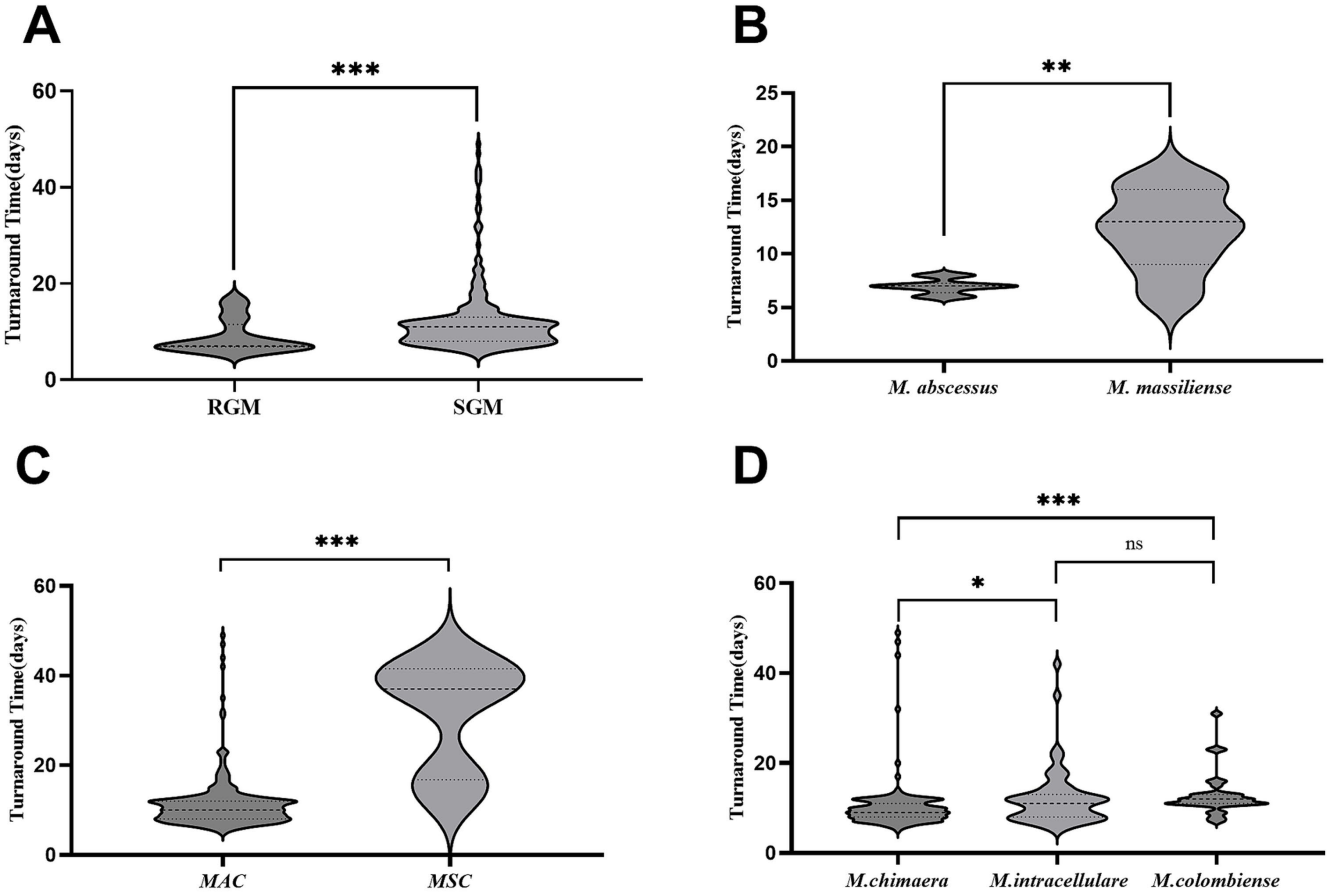
TAT comparisons among bacterial species. **(A)** TAT comparisons for RGM and SGM. **(B)** TAT comparisons for *M. abscessus* and *M. massiliense*. **(C)** TAT comparisons for MAC and MSC. **(D)** TAT comparisons for *M. chimaera*, *M. intracellulare*, and *M. colombiense*. Data are presented as medians, quartiles, maximums, and minimums. Data were compared among independent groups using the Mann–Whitney *U* test (two groups) and the Kruskal–Wallis test (multiple groups), followed by multiple comparisons using Dunn’s *post-hoc* test. *p < 0.05, **p < 0.01, ***p < 0.001, ns, not significant. TAT, turnaround time; RGM, rapidly growing mycobacteria; SGM, slow-growing mycobacteria; MAC, *Mycobacterium avium* complex; MSC, *Mycobacterium simiae* complex.

The median TAT for RGM was 7 days [interquartile range (IQR): 6.9–11.5], while the median TAT for SGM was 11 days (IQR: 8–13), representing a statistically significant difference (*p* < 0.001) (Figure 1A). Among the RGM, the median TAT for *M. abscessus* (MAB) was 7 days (IQR: 6.4–7.3), which was significantly shorter than that for *Mycobacterium massiliense*, which was 13 days (IQR: 9–16) (*p* = 0.005) (Figure 1B). Among SGM, the median TAT for MAC was 10 days (IQR: 8–12), which was significantly shorter than that for MSC, which was 37 days (IQR: 16.8–41.5) (*p* < 0.001) (Figure 1C). Within the MAC group, the median TAT for *M. chimaera* was 9 days (IQR: 8–11), which was shorter than that for *M. intracellulare* (11 days, IQR: 8–13; *p* = 0.015) and *M. colombiense* (12 days, IQR: 11–13; *p* < 0.001). However, there was no statistically significant difference between the median TATs of *M. intracellulare* and *M. colombiense* (*p* = 0.166) (Figure 1D).

### 3.3 Clinical factors related to TAT

TAT values varied significantly among different patient populations. Patients with NTM-PD had a median TAT of 8 days (IQR: 7–10), those with bronchiectasis had a median TAT of 9 days (IQR: 7.25–12), and those with lung cavitation had a median TAT of 8 days (IQR: 7–10). These groups exhibited shorter TATs compared with patients without these conditions (*p* < 0.05) (Table 2). Additionally, a negative correlation was observed between AFB smear grade and TAT (Spearman’s correlation coefficient = −0.49, *p* < 0.001) (Table 3).

**Table 2:** Comparison of the TAT across different populations.

Features	Variable (number)	Median TAT (IQR), day	*Z*-value	*p*-value
Sex	Male (154)	10.25 (8–12.25)	−0.053	0.958
	Female (135)	10 (8–13)		
Age	≤60 (48)	10 (9–13)		0.514
	61–70 (80)	12.25 (8–12.75)		
	71–80 (109)	10 (7.5–12)		
	>80 (51)	12 (8–13)		
Smoke history	Yes (143)	11 (8–13)	−1.066	0.287
	No (146)	10 (8–12)		
Tuberculosis history	Yes (33)	9 (7.25–12)	−1.919	0.055
	No (236)	11 (8–13)		
NTM-PD	Yes (71)	8 (7–10)	−5.66	<0.001
	No (278)	11 (9–13)		
HIV	Yes (20)	10 (7.25–12)	−0.762	0.446
	No (269)	10 (8–13)		
COPD	Yes (55)	11 (8–13)	−1.105	0.269
	No (234)	10 (8–12)		
Bronchiectasis	Yes (81)	9 (7.25–12)	−2.658	0.008
	No (208)	11 (8–13)		
Pulmonary cavity	Yes (55)	8 (7–10)	−5.03	<0.001
	No (234)	11 (9–13)		
Lung nodules	Yes (36)	10.5 (8–12)	−0.09	0.928
	No (253)	10 (8–13)		
Tumor	Yes (56)	1 1 (8–13)	−0.652	0.514
	No (233)	10 (8–12)		
Pulmonary surgery	Yes (17)	9 (7–13)	−0.778	0.436
	No (272)	10 (8–12.75)		
Albumin	Normal (72)	10 (8–12)	−0.003	0.998
	Low (278)	10 (8–13)		
Antinuclear antibodies	Positive (68)	10 (8–12.75)	−0.3	0.764
	Negative (96)	10.75 (9–13)		
Rheumatoid factor	Abnormal (28)	10.5 (7.6–12)	−0.92	0.358
	Normal (115)	11 (9–13)		

Comparisons of the TAT between independent groups were performed using the Mann–Whitney *U* test (two groups) and the Kruskal–Wallis test (multiple groups).

TAT, turnaround time; IQR, interquartile range; NTM-PD, NTM pulmonary disease; COPD, chronic obstructive pulmonary disease.

**Table 3:** Correlation between the AFB smear grade and the TAT via Spearman’s correlation analysis.

Microscopic smear*	Median TAT (IQR), day
Negative (*N* = 208)	11 (9–13)
Few (*N* = 16)	7.5 (6.1–8)
1 + (*N* = 13)	8 (7–9)
2 + (*N* = 13)	7 (6–8)
3 + (*N* = 9)	7 (7–8)
4 + (*N* = 2)	7.25

^*^Few (1–8/300 fields), 1 + (3–9/100 fields), 2 + (1–9/10 fields), 3 + (1–9/field), and 4 + (≥10/field).

Spearman’s correlation coefficient = −0.49; *p*-value <0.001.

TAT, turnaround time; AFB, acid-fast bacilli; IQR, interquartile range.

### 3.4 Independent factors influencing TAT

In the generalized linear model, the AFB smear result was the only independent factor significantly associated with TAT (Wald *χ*^2^ = 39.71, *p* < 0.001). The incidence rate ratio was 0.61 (95% CI: 0.52–0.71), indicating that positive AFB smears were associated with a 39% reduction in TAT. Other variables, including mycobacterial species, NTM-PD, pulmonary cavity, and bronchiectasis, did not show significant independent effects (all *p* > 0.05) (Table 4).

**Table 4:** Independent factors associated with TAT identified using the multivariate generalized linear model.

Variable	Coefficient (B)	IRR (95% CI)	*p*-value
AFB smear (positive vs. negative)	−0.493	0.61 (0.52–0.71)	<0.001
Mycobacterial species (RGM vs. SGM)	−0.098	0.91 (0.74–1.11)	0.346
NTM-PD (yes vs. no)	0.067	1.07 (0.93–1.23)	0.340
Pulmonary cavity (yes vs. no)	−0.046	0.96 (0.84–1.09)	0.521
Bronchiectasis (yes vs. no)	−0.068	0.93 (0.84–1.04)	0.230

A gamma distribution with log link function was used. The overall model was statistically significant (omnibus test *χ*^2^ = 56.70, df = 5, *p* < 0.001). A *p*-value of < 0.05 was considered statistically significant.

TAT, turnaround time; IRR, incidence rate ratio; AFB, acid-fast bacilli; RGM, rapidly growing mycobacteria; SGM, slow-growing mycobacteria; NTM-PD, NTM pulmonary disease.

## 4 Discussion

Advancements in technology have identified more than 200 types of NTM, but the primary pathogens of NTM disease include MAC, MABC, and *Mycobacterium kansasii* (13, 14). MABC, which is part of the RGM, typically grows within 3–5 days, whereas MAC, a member of the SGM, requires 1 week or more to grow. Our results align with these observations, with a median TAT of 7 days for MABC and 10 days for MAC, which is consistent with the TTP values reported by Uwamino et al. (15). These findings suggest that TAT reflects strain growth rates, providing useful diagnostic insights.

MABC includes the subspecies MAB, *M. massiliense*, and *Mycobacterium bolletii*, which differ in drug susceptibility, making subspecies identification crucial for treatment (15). Our data indicate that MAB grows faster than *M. massiliense* does, allowing TAT to distinguish between these subspecies—a feature not previously emphasized in the literature. Similarly, TAT can differentiate *Mycobacterium chimaera* from other MAC subspecies, despite an earlier report suggesting no differences in TTP among MAC members (16). However, the previous study involved smaller sample sizes, potentially introducing bias. Our findings, supported by a larger dataset, highlight the potential of TAT for distinguishing these strains.

MSC, a large complex within the NTM, contains approximately 20 strains (17), which exhibit varying growth requirements (18–20). Despite being SGM, the calculated TAT for MSC was significantly longer than that for MAC. This discrepancy may stem from the limited number of MSC cases (*n* = 6) in our study, which included only *Mycobacterium lentiflavum* and *Mycobacterium paraense*, potentially biasing the results. Another explanation could be the rarity of MSC-associated NTM-PD (21), which complicates isolation and prolongs TAT.

In our study, TAT was significantly correlated with NTM-PD and bronchiectasis. Patients with NTM-PD require at least two positive sputum samples or one positive lavage sample for diagnosis (22). This diagnostic criterion implies a greater bacterial load in the respiratory tract, leading to shorter TTPs, higher AFB smear grades, and consequently shorter TATs. Our data confirmed a strong correlation between TAT and AFB smear grade, and the acid-fast smear result was identified as the key independent factor influencing TAT. Notably, acid-fast bacilli (AFB) smear results were categorized into six grades in the correlation analysis (Table 3). In the generalized multiple linear regression model, however, the AFB smear results were dichotomized into negative and positive (few to 4+) to facilitate clinical interpretation and ensure model stability (Table 4). Although bronchiectasis is not a common susceptibility factor for tuberculosis (13), it is closely associated with NTM disease (23, 24). Conversely, despite previous reports linking NTM disease to underlying conditions such as tuberculosis, chronic obstructive pulmonary disease, and autoimmune diseases (25), our data did not suggest an association between these conditions and the TAT. We hypothesize that the bacterial load in samples has a more direct impact on TAT than the underlying conditions.

According to the American Thoracic Society and the Infectious Diseases Society of America, NTM-PD diagnosis requires a combination of clinical, imaging, and microbiological findings (22, 26). The imaging criteria included nodules or cavitations in the lungs (26). Our data suggest that lung cavitation affects the TAT more than pulmonary nodules do, which may be due to the lack of distinction between nodule size and quantity in our analysis. Additionally, the median TAT in the population with pulmonary cavities was 8 days in our study, which contrasts with the previously reported TTP of 12 days in a smaller cohort (*n* = 12) (10), suggesting that our larger sample size (*n* = 55) provides more reliable estimates.

The TTP for the BACTEC MGIT 960 system, which uses an oxygen-quenching fluorescence sensor and a specific algorithm to detect culture positivity (27), has been shown to correlate negatively with AFB smear grade (9, 10), and pretreatment TTP correlates negatively with treatment response (28). Our findings further revealed that TAT correlates with bacterial load, suggesting its potential as a biomarker for therapeutic efficacy, although further research is needed.

Notably, given the limited number of TAT studies in the NTM, we only compared TAT with the TTP in previous literature. The TAT in this study was reduced by 2–3 days to calculate the corresponding TTP. However, there were a small number of cases (*n* = 6) in which we sequenced the specimens before issuing the report due to unclear smear morphology or findings that were inconsistent with historical results. Therefore, the TAT for these samples was prolonged. In addition, compared with the TTP, the TAT is easily accessible and can be obtained directly from the report sheet (12). The Chinese Basic Technical Standards for Clinical Microbiology Testing (WS/T805-2022) mandates that the laboratory is obligated to report the TAT of the sample. Thus, our exploration of TAT provides valuable insights for the clinical management of NTM.

As an indicator reflecting laboratory efficiency (6, 12), we recommend that each laboratory using the BACTEC MGIT 960 System establishes species-specific TAT benchmarks based on its own circumstances. For instance, the TAT benchmark for RGM could be set at 7–10 days and that for MAC at 10–14 days. When the actual TAT exceeds the benchmark, the laboratory can promptly investigate potential issues (e.g., sample quality, instrument status, or operational errors) to improve testing efficiency.

There are several limitations in this study, despite the meaningful findings. First, this was a retrospective study conducted in a single hospital, so the results might vary in other regions due to heterogeneity in species composition. Second, the sample size of RGM is significantly smaller than that of SGM, which constitutes an inherent limitation of the present research. Third, TAT in this study does not include species identification because species identification is not routinely performed in our clinical laboratory. In the future, the collection of RGM strains from multicenter collaboration will further verify the generalizability and robustness of our conclusions.

In summary, the TAT of NTM varies significantly by species. Patients with NTM-PD, bronchiectasis, or lung cavitation exhibit shorter TATs. Additionally, the AFB smear result was identified as the only independent factor significantly associated with TAT in the generalized linear model. These findings can be helpful in optimizing the laboratory detection workflow of mycobacteria and reasonably predicting the detection cycle. They may also provide support for clinical decision-making among physicians.

## Data Availability

The original contributions presented in the study are included in the article/supplementary material, further inquiries can be directed to the corresponding author.
